# Academic Outcomes Following Implementation of Case Scenario‐Based Teaching in Postgraduate Health Promotion Education: A Repeated‐Cohort Study, 2019–2025

**DOI:** 10.1002/hpja.70279

**Published:** 2026-11-05

**Authors:** Hassan Hosseingzadeh, Joseph Edwards, Jamila Butayeva, Zubair Ratan, Marzieh Niknami, Iksheta Verma, Ann Dadich

**Affiliations:** ^1^ Faculty of Science, Medicine and Health, School of Medical, Indigenous and Health Sciences University of Wollongong Wollongong Australia; ^2^ Remote Health Systems and Climate, Change Centre, Menzies School of Health Research Alice Springs Australia; ^3^ Highlands General Practice Bowral New South Wales Australia; ^4^ School of Medicine – Deakin University Melbourne Victoria Australia; ^5^ Business School Western Sydney University Penrith New South Wales Australia

## Abstract

**Issue Addressed:**

Case scenario‐based teaching (CSBT) is used to support applied and contextualised learning in health promotion education, but evidence on academic outcomes across successive cohorts is limited.

**Methods:**

This repeated‐cohort observational study examined de‐identified secondary data from 765 postgraduate public health students at the University of Wollongong, Australia, between 2019 and 2025. Cohort differences were assessed using chi‐square tests, Welch's analysis of variance with Games–Howell comparisons, generalised linear models with robust covariance estimates and adjusted binary logistic regression. The 2019 cohort was treated as the reference cohort.

**Results:**

Most participants were female (72.9%), aged 24–29 years (53.2%) and international students (62.1%). Mean total marks differed across cohorts (Welch *F* (6, 297.95) = 17.19, *p* < 0.001); Games–Howell comparisons with 2019 were significant for 2020, 2021, 2024 and 2025, but not 2022 or 2023. After adjustment for gender, age group, enrolment and delivery mode, cohort year remained associated with total marks (Wald *χ* ^2^ (6) = 35.20, *p* < 0.001). Grade distribution also differed by year (*χ* ^2^ (24) = 218.63, *p* < 0.001). In the adjusted Distinction/High Distinction model, cohort year was significant overall (Wald *χ* ^2^ (6) = 22.98, *p* < 0.001), although only the 2021 contrast with 2019 was individually significant.

**Conclusions:**

Academic outcomes varied across successive cohorts in a subject using CSBT, and several later cohorts had higher total marks than the 2019 reference cohort after adjustment for measured student characteristics. Because this was a repeated‐cohort observational study with concurrent curriculum and assessment changes, the findings describe temporal associations and do not establish a causal effect of CSBT.

## 1 Introduction

Didactic teaching, where students primarily acquire knowledge passively, has been deemed ill‐suited to the current, fast‐evolving educational environment, which prioritises the development of skills in critical thinking, communication, collaboration and creativity [1]. Meta‐analyses have found that integrating critical thinking and problem‐based learning improves cognitive abilities, collaborative skills and academic performance [2, 3]. Case scenario‐based teaching (CSBT) addresses this gap. A prior study at the University of Wollongong, Australia, reported immediate educational changes among Master of Public Health students exposed to CSBT in the 2019 cohort [4]. That study focused on the initial cohort and immediate educational outcomes. The present study extends that work by examining routinely collected academic outcomes across successive cohorts from 2019 to 2025, with 2019 used as the reference cohort. Because only one reference‐year observation was available and there was no concurrent control group, the present evaluation was designed as a repeated‐cohort observational study rather than an interrupted time‐series analysis.

CSBT is a pedagogical approach in which ‘cases’ present hypothetical or real‐world scenarios as context, enabling students to engage in active learning that promotes their problem‐solving and critical‐thinking skills [5]. To achieve this, each case study is structured to mirror the complex interplay of socio‐economic and political forces and competing interests that can impede health priorities [6]. This can enable students to consider multiple perspectives in collaboration with team members and work towards joint decisions. This pedagogical approach favours critical thinking over linear thinking, empowering students to weigh facts as evidence, interrogate assumptions and examine multiple perspectives in the decision‐making process in collaboration with team members. CSBT has been the hallmark of curriculum development and student assessment in teaching health promotion subjects at the University of Wollongong since the publication of the 2019 baseline study [4].

Several factors underscore the importance of CSBT in diverse higher education settings. The top 10 countries have consistently been from Asia and South America, accounting for more than 70% of international students in Australia across all levels of education since 2019. China, India and Nepal have retained the top three positions, while Bangladesh, the Philippines, Pakistan and Sri Lanka were among the top 10 in 2025 [7]. The representation of international students increased from 32.4% to 35.2% in 2024 [8]. Given the growing diversity and proportion of international students in higher education, universities need teaching methods that improve learning outcomes across diverse linguistic, cultural and educational backgrounds [9]. CSBT can be useful in this context as it involves developing inclusive classroom settings, discussing multiple perspectives, providing equitable access to education and facilitating improved academic performance among diverse student cohorts [10].

CSBT can also improve human capital, as it ultimately promotes productivity and profitability in the workforce [11]. The development of social capital has been associated with the sharing of experiences and innovative ideas [12], which are the main focuses of CSBT. Furthermore, the effectiveness of human capital is best demonstrated by its ability to adapt to a dynamic environment and emerging technologies in the real world [13]. However, a persistent significant challenge is the gap between knowledge acquired and its application in real‐world workplace settings. CSBT can bridge this gap by providing students with opportunities to apply knowledge to uncertain situations before joining the workforce.

Importantly, CSBT provides a rich and shared group‐learning environment enabling students to practise complex reasoning before entering professional workplace settings. This can enhance employability competencies development, including knowledge retention, confidence, collaboration and evidence‐based judgement, through frequent engagement with contextual data, peer discussion and the reconciliation of differences in an encouraging and supportive learning group environment [5, 14]. This approach appears to be particularly attractive for international students, as CSBT can enable peer interaction and contextualised learning, bolstering participation, confidence, critical thinking and learning in diverse cultural learning environments [15]. Although language difficulties can be challenging for many international students [16], the context‐based approach embedded in CSBT retains students' interest in expressing their own perspectives. Furthermore, given its team‐based format, CSBT can provide a less intimidating environment for international students to express opinions and contribute to the case study assessment. In this respect, CSBT may provide a more inclusive learning platform for domestic and international students by offering opportunities for engagement, peer learning, collaboration and applied reasoning.

Despite the growing use of CSBT and case‐based learning approaches in health promotion, public health and medical education [17, 18], evidence on academic outcomes across successive cohorts remains limited. This repeated‐cohort observational study therefore examined patterns in total marks and grade distributions among postgraduate public health students from 2019 to 2025, using the 2019 cohort as the reference cohort. The study also examined whether observed cohort differences persisted after adjustment for measured student characteristics. The findings aim to inform health promotion teaching and curriculum design; they are not intended to establish a causal effect of CSBT or to infer competencies that were not directly measured.

## 2 Methods

### 2.1 Research Design, Participants and Procedure

A retrospective repeated‐cohort observational study was conducted at the University of Wollongong, Australia. The study examined data from a health promotion subject delivered to postgraduate public health students between 2019 and 2025. The subject was HAS948 Health Promotion, which was renamed PGPH903 Health Economics, Policy and Promotion in 2023 following public health course restructuring. It was offered online and on‐campus. The study used de‐identified secondary data routinely collected from 765 postgraduate students enrolled in the subject. The data were derived from the University of Wollongong student records (i.e., Student Management Package). All personal identifiers including names and student numbers were removed before data analysis. This study was reviewed and approved by the Health and Medical Human Research Ethics Committee at the University of Wollongong (Ethics Number: H2026‐0120). The CSBT approach and its theoretical basis were previously described [4]. Briefly, unfolding and incomplete scenarios encouraged students to identify problems, evaluate relevant information, examine contextual factors, apply health promotion theories, develop alternative solutions and justify recommendations. Case scenarios reflected real‐world health promotion challenges and were embedded in lectures, study materials, tutorials and assessments. The 2019 cohort was used as the reference cohort for comparisons across successive annual cohorts.

Across the study period, the subject coordinator remained the same, the main teaching team was broadly stable and the core learning outcomes, marking and moderation processes and rubrics for comparable tasks were substantially unchanged. The subject was designed for both online and on‐campus delivery; during COVID‐related restrictions it was delivered online only. The intended scope and intensity of CSBT were stable across the study period. In contrast, the assessment mix changed, particularly following the 2023 subject restructure; these contextual changes were considered in the analyses and interpretation.

### 2.2 Study Measures

#### 2.2.1 Demographics and Academic Characteristics

Demographics and academic attributes included gender, age, country of origin, subject delivery modes, enrolment type and enrolment year.

#### 2.2.2 Assessment and Total Marks

The subject had four assessments (2019, 2020, 2021, 2022 and 2025) or five assessments (2023 and 2024) that totalled 100 marks. The assessments included a health promotion essay, report, examination, health policy essay, quiz, health promotion economics evaluation essay, group project, health policy essay and reflection (see Table 2). The health promotion essay (weight: 10%, 2019–2022) was a 500‐word essay requiring students to discuss the application of the Ottawa charter [19] for health promotion to current health challenges using three supporting and one counter arguments. The report (weight: 30%, 2019–2022), a 1500‐word assessment, required students to choose a health promotion issue; examine four interventions implemented to change individual behaviours; and determine the most effective intervention using scholarly literature. The examination (weight: 40%, 2019–2022) was a 2‐h assessment, which included two case scenarios and 20 multiple‐choice items. Students were required to examine the impact of globalisation on health promotion; apply a health promotion perspective in various real‐world settings and evaluate the significance of different theories in health promotion interventions. A reflection (weight: 20%, 2019–2025) consisted of fortnightly tutorial activities, requiring students to discuss case scenarios within small groups of 5–8 students to: promote different understandings; present and debate ideas; discuss the strengths and weaknesses of health promotion initiatives offered by group members and adjust or modify their ideas at the end of sessions within their group. Contribution to each tutorial session was evaluated using a modified valid rubric with 4 criteria including frequency of participation, quality of comments, listening skills and level of preparation in class [20]. The scale ranged from 1 to 4 for each criterion, with 4 indicating the highest score. A health policy essay (weight: 20% in 2023–2024 and 25% in 2025) took the form of an 800‐word assessment, whereby students were required to examine an Australian health policy, its objectives, context, impacts, strengths, weaknesses and future directions. A quiz (weight: 5% in 2023–2024 and 15% in 2025) assessed students' knowledge of health economics including net benefit, opportunity cost, shadow price, threshold value, equity and performance measures. A health promotion economics evaluation essay (weight: 20%, 2023–2024) involved an 800‐word assessment, requiring students to demonstrate sound knowledge of robust methods for health promotion health economics evaluation. A group project (weight: 35% in 2023–2024 and 40% in 2025) required students to critically analyse a published peer‐reviewed journal article about a health promotion intervention and present their findings as a group presentation. It examined students' skills and ability to explain the intervention, evaluate its theoretical foundations, assess its appropriateness for the target population, evaluate its strengths and limitations and suggest improvements informed by health promotion principles including supportive environments, healthy public policy, personal skills, community action and health services reorientation. The total mark was determined by adding up the weighted marks gained across all assessments offered each year. The reflection assessment was the only component that remained unchanged across 2019–2025 in weighting (20%), format, instructions, rubric and grading approach. It was therefore analysed separately as a stable assessment outcome. Because the remaining assessment mix changed over time, total marks were interpreted within each year's assessment architecture.

#### 2.2.3 Grades

Student grades were calculated based on their total marks. They received a Fail grade if they obtained < 50% of the total mark; a pass for 50%–64% of the total mark; a credit for 65%–74% of the total mark; a distinction for 75%–84% of the total mark or a high distinction for 85%–100% of the total mark.

### 2.3 Data Analysis

The unit of analysis was an individual student enrolment record. Descriptive statistics summarised demographic and academic characteristics, assessment outcomes, total marks and grades. Year was modelled as a categorical variable, with 2019 as the reference cohort. Differences in cohort composition by year were examined using Pearson chi‐square tests with Cramer's *V*. Because homogeneity of variance was violated for total marks and Reflection scores (Levene tests *p* < 0.001), cohort differences in these continuous outcomes were assessed using Welch's one‐way analysis of variance followed by Games–Howell comparisons with the 2019 reference cohort. The stable Reflection assessment was analysed separately as a sensitivity outcome. Adjusted total marks were examined using generalised linear models with a normal distribution, identity link and robust covariance estimates. The primary adjusted model included year, gender, age group, enrolment type and delivery mode; Bonferroni‐adjusted estimated marginal mean contrasts compared each cohort with 2019. A sensitivity model replaced enrolment type with country/region because of conceptual overlap between enrolment and country. For adjusted grade analyses, grades were dichotomised as Distinction/High Distinction versus Fail/Pass/Credit and analysed using binary logistic regression with the same covariates; a corresponding country/region sensitivity model was also fitted. Model diagnostics included inspection of standardised Pearson residuals and residual‐versus‐predicted plots for continuous‐outcome models and the Hosmer–Lemeshow goodness‐of‐fit test for logistic models. Sixteen observations with standardised Pearson residuals exceeding ±3 were reviewed and represented genuine low final marks, predominantly among students who remained enrolled but did not complete all assessment tasks; these observations were retained. All 765 records had complete data for variables included in the final models, and no imputation was performed. Tests were two‐sided with *p* < 0.05 and 95% confidence intervals. Analyses were conducted using IBM SPSS Statistics, version 31.

## 3 Results

### 3.1 Demographics

A total of 765 postgraduate public health student enrolment records were included (Table 1). Most students were female (72.9%), aged 24–29 years (53.2%), international (62.1%) and enrolled in on‐campus delivery (66.9%); 50.1% were from South Asia. Cohort composition varied significantly by year for gender (*χ* ^2^ (6) = 12.93, *p* = 0.044, Cramer's *V* = 0.130), age group (*χ* ^2^ (24) = 77.15, *p* < 0.001, V = 0.159), country/region (*χ* ^2^ (18) = 123.27, *p* < 0.001, *V* = 0.232), enrolment type (*χ* ^2^ (6) = 114.49, *p* < 0.001, *V* = 0.387) and delivery mode (*χ* ^2^ (6) = 124.49, *p* < 0.001, *V* = 0.403). These differences supported adjustment for measured cohort characteristics in subsequent models.

**TABLE 1:** Participant demographic and academic variables (*N* = 765).

Variables	% (*n*)
Gender	
Male	27.1% (207)
Female	72.9% (558)
Age group	
Early adulthood (22–23 years)	9.9% (76)
Prime young adult learners (24–29 years)	53.2% (407)
Early career adults (30–35 years)	20.9% (160)
Mid‐age learners (36–45 years)	11.4% (87)
Mature learners (46+ years)	4.6% (35)
Country	
Australia	32.0% (245)
South Asia (India, Nepal, Pakistan and Bangladesh)	50.1% (383)
Asia–Pacific (excluding South Asia)	8.9% (68)
Rest of world (Europe, Africa and Americas)	9.0% (69)
Enrolment	
Domestic	37.9% (290)
International	62.1% (475)
Delivery	
Online	33.1% (253)
On‐campus	66.9% (512)
Subject	
HAS948	54.2% (415)
PGPH903	45.8% (350)
Enrolment year	
2019	18.0% (138)
2020	17.0% (130)
2021	8.4% (64)
2022	10.8% (83)
2023	21.3% (163)
2024	11.9% (91)
2025	12.5% (96)

### 3.2 Types of Assessment and Distribution

The assessment architecture changed over time, particularly after the 2023 subject restructure (Table 2), so scores from different assessment types were not treated as directly comparable. Reflection was the only assessment that remained unchanged in weighting, format, instructions, rubric and grading across all cohorts. Mean Reflection scores differed significantly across cohorts (Welch *F* (6, 299.24) = 28.26, *p* < 0.001). Games–Howell comparisons showed higher Reflection scores in every cohort from 2020 to 2025 compared with 2019 (all *p* ≤ 0.004). These scores represent performance on the Reflection assessment and were not interpreted as direct measures of engagement, teamwork, or other competencies.

**TABLE 2:** Assessment structure and reflection scores across student cohorts, 2019–2025 (*N* = 765).

Year	Assessment structure and weighting	Reflection (20%), *M* (SD)
2019 (*N* = 138)	Health promotion essay 10%; report 30%; examination 40%; reflection 20%	12.94 (3.06)
2020 (*N* = 130)	Health promotion essay 10%; report 30%; examination 40%; reflection 20%	15.06 (4.56)
2021 (*N* = 64)	Health promotion essay 10%; report 30%; examination 40%; reflection 20%	14.95 (3.66)
2022 (*N* = 83)	Health promotion essay 10%; report 30%; examination 40%; reflection 20%	16.54 (3.21)
2023 (*N* = 163)	Health policy essay 20%; quiz 5%; health promotion economics essay 20%; group project 35%; reflection 20%	16.94 (3.72)
2024 (*N* = 91)	Health policy essay 20%; quiz 5%; health promotion economics essay 20%; group project 35%; reflection 20%	16.56 (3.32)
2025 (*N* = 96)	Health policy essay 25%; quiz 15%; group project 40%; reflection 20%	17.02 (2.62)

Abbreviations: *M* = mean, SD = standard deviation.

### 3.3 Total Mark and Academic Grade Distribution

Mean total marks differed significantly across cohorts (Welch *F* (6, 297.95) = 17.19, *p* < 0.001; Table 3). Games–Howell comparisons with the 2019 reference cohort showed higher mean total marks in 2020 (mean difference 9.75, 95% CI 3.52 to 15.97, *p* < 0.001), 2021 (16.33, 95% CI 9.30–23.35, *p* < 0.001), 2024 (11.08, 95% CI 5.71–16.46, *p* < 0.001) and 2025 (9.39, 95% CI 4.28–14.51, *p* < 0.001). Differences for 2022 and 2023 were not statistically significant. Grade distribution also differed by year (*χ* ^2^ (24) = 218.63, *p* < 0.001). Fail grades declined from 16.7% in 2019 to 2.1% in 2025, while the proportion achieving Distinction/High Distinction varied non‐linearly across cohorts, peaking at 67.2% in 2021, falling in 2022–2023, increasing in 2024 and reaching 32.3% in 2025.

**TABLE 3:** Total marks and academic grade distribution across student cohorts, with comparisons to the 2019 reference cohort (*N* = 765).

Year	Total mark, *M* (SD)	Difference vs. 2019 (95% CI), p	Fail % (*n*)	Pass % (*n*)	Credit % (*n*)	Distinction % (*n*)	High Distinction % (*n*)	Overall grade *χ* ^2^
2019 (*N* = 138)	61.1 (17.2)	Reference	16.7% (23)	35.5% (49)	22.5% (31)	20.3% (28)	5.1% (7)	
2020 (*N* = 130)	70.8 (17.1)	+9.75 (3.52–5.97), *p* < 0.001	8.5% (11)	15.4% (20)	25.4% (33)	30.0% (39)	20.8% (27)	
2021 (*N* = 64)	77.4 (14.7)	+16.33 (9.30–23.35), *p* < 0.001	3.1% (2)	7.8% (5)	21.9% (14)	28.1% (18)	39.1% (25)	
2022 (*N* = 83)	63.6 (11.6)	+2.52 (−3.27–8.31), *p* = 0.853	4.8% (4)	47.0% (39)	26.5% (22)	16.9% (14)	4.8% (4)	
2023 (*N* = 163)	64.6 (10.5)	+3.49 (−1.52–8.50), *p* = 0.372	1.8% (3)	46.6% (76)	27.0% (44)	22.7% (37)	1.8% (3)	
2024 (*N* = 91)	72.1 (10.1)	+11.08 (5.71–6.46), *p* < 0.001	3.3% (3)	13.2% (12)	38.5% (35)	37.4% (34)	7.7% (7)	
2025 (*N* = 96)	70.5 (8.8)	+9.39 (4.28–14.51), *p* < 0.001	2.1% (2)	17.7% (17)	47.9% (46)	30.2% (29)	2.1% (2)	
								*χ* ^2^ (24) = 218.63, *p* < 0.001

*Note:* Grading scale: Fail (0%–49%), Pass (50%–64%), Credit (65%–74%), Distinction (75%–84%) and High Distinction (85%–100%).

Differences in mean total marks across cohorts were assessed using Welch's ANOVA followed by Games–Howell post hoc comparisons. Comparisons shown are against the 2019 reference cohort. Grade distributions were compared using Pearson's chi‐square test.

Abbreviations: CI = confidence interval, *M* = mean, SD = standard deviation.

### 3.4 Associations Between Student Characteristics and Academic Grades

Unadjusted grade‐category associations are shown in Table 4. Gender was not significantly associated with the three‐category grade outcome (*χ* ^2^ (2) = 5.85, *p* = 0.054, Cramer's *V* = 0.087). Significant associations were observed for age group (*χ* ^2^ (8) = 30.39, *p* < 0.001, *V* = 0.141), country/region (*χ* ^2^ (6) = 221.06, *p* < 0.001, *V* = 0.380), enrolment type (*χ* ^2^ (2) = 179.97, *p* < 0.001, *V* = 0.485) and delivery mode (*χ* ^2^ (2) = 117.77, *p* < 0.001, *V* = 0.392). Distinction/High Distinction was observed in 65.5% of domestic compared with 17.7% of international students, and in 62.5% of online compared with 22.7% of on‐campus students. These are unadjusted associations and were interpreted alongside the adjusted models.

**TABLE 4:** Associations between student characteristics and academic grade category (*N* = 612).

Characteristic	Category	Fail *n* (%)	Pass/Credit *n* (%)	Distinction/HD *n* (%)	*χ* ^2^ (df), *p*; Cramer's *V*
Gender	Male	17 (8.2)	129 (62.3)	61 (29.5)	5.85 (2), *p* = 0.054; *V* = 0.087
	Female	31 (5.6)	314 (56.3)	213 (38.2)	
Age group	22–23 years	4 (5.3)	33 (43.4)	39 (51.3)	30.39 (8), *p* < 0.001; *V* = 0.141
	24–29 years	36 (8.8)	255 (62.7)	116 (28.5)	
	30–35 years	6 (3.8)	85 (53.1)	69 (43.1)	
	36–45 years	1 (1.1)	52 (59.8)	34 (39.1)	
	46+ years	1 (2.9)	18 (51.4)	16 (45.7)	
Country/region	Australia	7 (2.9)	66 (26.9)	172 (70.2)	221.06 (6), *p* < 0.001; *V* = 0.380
	South Asia	37 (9.7)	298 (77.8)	48 (12.5)	
	Asia–Pacific	1 (1.5)	38 (55.9)	29 (42.6)	
	Rest of world	3 (4.3)	41 (59.4)	25 (36.2)	
Enrolment	Domestic	7 (2.4)	93 (32.1)	190 (65.5)	179.97 (2), *p* < 0.001; *V* = 0.485
	International	41 (8.6)	350 (73.7)	84 (17.7)	
Delivery	Online	6 (2.4)	89 (35.2)	158 (62.5)	117.77 (2), *p* < 0.001; *V* = 0.392
	On‐campus	42 (8.2)	354 (69.1)	116 (22.7)	

*Note:* Percentages are calculated within each characteristic category. Grade categories were grouped as Fail, Pass/Credit and Distinction/High Distinction. Associations were assessed using Pearson's chi‐square test; effect size is reported using Cramer's *V*.

### 3.5 Adjusted Analyses

After adjustment for gender, age group, enrolment type and delivery mode, cohort year remained associated with total marks (Wald *χ* ^2^ (6) = 35.20, *p* < 0.001; Table 5). Compared with 2019, Bonferroni‐adjusted contrasts were significant for 2020 (adjusted difference 5.40, *p* = 0.038), 2021 (10.14, *p* < 0.001), 2024 (7.16, *p* < 0.001) and 2025 (5.04, *p* = 0.018), but not for 2022 or 2023. A sensitivity model replacing enrolment with country/region showed a similar overall year association (Wald *χ* ^2^ (6) = 31.99, *p* < 0.001), although the 2020 contrast was attenuated and no longer significant. In the adjusted binary logistic model for Distinction/High Distinction, year was significant overall (Wald *χ* ^2^ (6) = 22.98, *p* < 0.001), but only the 2021 cohort differed significantly from 2019 (OR 2.19, 95% CI 1.04–4.59, *p* = 0.039); all other year‐specific contrasts were non‐significant. Model fit was acceptable (Hosmer–Lemeshow *p* = 0.264). The country/region sensitivity model produced the same overall interpretation (year Wald *χ* ^2^ (6) = 18.11, *p* = 0.006), with 2021 remaining the only significant year‐specific contrast (OR 2.64, 95% CI 1.22–5.68, *p* = 0.013).

**TABLE 5:** Adjusted associations between cohort year and academic outcomes (*N* = 765).

Year	Adjusted total mark mean	95% CI	Bonferroni *p* vs. 2019	Adjusted OR for D/HD	95% CI	*p*
2019	65.03	62.01–68.05	Reference	1.00	Reference	—
2020	70.44	67.60–73.28	0.038	1.43	0.78–2.62	0.249
2021	75.18	71.57–78.78	< 0.001	2.19	1.04–4.59	0.039
2022	66.23	63.62–68.84	1.000	0.56	0.27–1.16	0.120
2023	67.63	65.83–69.42	0.577	0.72	0.40–1.30	0.282
2024	72.19	69.99–74.39	< 0.001	1.18	0.61–2.27	0.628
2025	70.08	67.93–72.22	0.018	0.53	0.27–1.04	0.064

*Note:* Primary adjusted models included cohort year, gender, age group, enrolment type and delivery mode. For total marks, generalised linear models used a normal distribution, identity link and robust covariance estimates; estimated marginal means are shown with Bonferroni‐adjusted contrasts versus 2019. For grades, binary logistic regression modelled Distinction/High Distinction versus Fail/Pass/Credit. Overall year effects: total marks Wald *χ* ^2^ (6) = 35.20, *p* < 0.001; D/HD Wald *χ* ^2^ (6) = 22.98, *p* < 0.001.

Abbreviations: CI = confidence interval, OR = odds ratio.

### 3.6 Descriptive Academic Outcomes Across Cohorts

Figure 1 presents unadjusted descriptive academic outcomes across cohorts. Mean total marks, the proportion achieving Distinction/High Distinction and the proportion receiving a Fail grade varied across years without a monotonic pattern. Mean total marks were higher than in the 2019 cohort in several subsequent years, although lower values were observed in 2022 and 2023. The proportion of students achieving Distinction/High Distinction increased markedly in 2020 and 2021, declined in 2022 and 2023, increased again in 2024 and decreased in 2025. In contrast, the proportion receiving a Fail grade was lower in every subsequent cohort than in 2019, although some year‐to‐year fluctuation remained.

**FIGURE 1:**
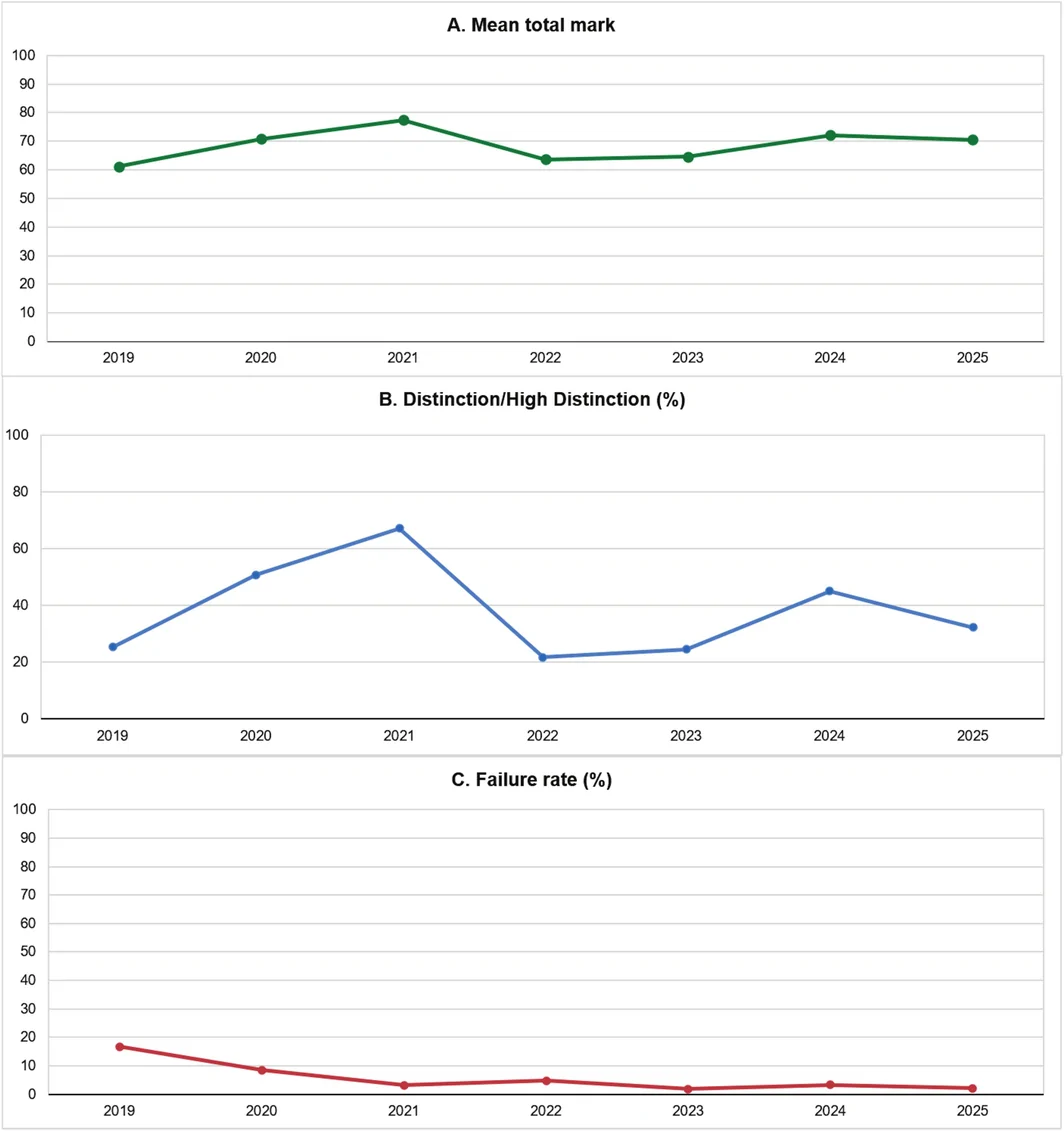
Unadjusted academic outcomes across student cohorts, 2019–2025. (A) Mean total mark; (B) proportion achieving Distinction or High Distinction and (C) proportion receiving a Fail grade.

## 4 Discussion

Health promotion education requires students to develop not only theoretical knowledge and understanding, but also the capacity to apply theoretical concepts to complex, real‐world health promotion challenges. Conventional didactic teaching approaches can limit the development of higher‐level analytical, interpretive, collaborative and problem‐solving skills [21, 22]. CSBT is an active learning approach that can provide opportunities to apply theory in contextualised scenarios [4, 5], and case‐based approaches are increasingly used in health and medical education [17, 18]. However, evidence on academic outcomes across successive health promotion cohorts is limited. This study aimed to address this gap by examining routinely collected academic outcomes among 765 postgraduate public health student enrolments from 2019 to 2025.

Academic outcomes varied across successive cohorts following the establishment of CSBT within the subject. Mean total marks were higher than in the 2019 reference cohort in 2020, 2021, 2024 and 2025, while 2022 and 2023 did not differ significantly. The adjusted total‐mark model showed a similar pattern after accounting for gender, age group, enrolment type and delivery mode. Grade distributions also differed across years, and Fail rates were lower in later cohorts; however, Distinction/High Distinction proportions followed a non‐linear pattern, and most adjusted year‐specific grade contrasts were reduced. As such, the findings indicate temporal associations across cohorts rather than a sustained causal effect attributable to CSBT.

Interpretation of total marks requires caution because the assessment mix changed over the study period. To address this, Reflection was analysed separately because it retained the same 20% weighting, task format, instructions, rubric and grading approach across all cohorts. Reflection scores were significantly higher in every cohort from 2020 to 2025 than in 2019. This sensitivity analysis suggests that the observed academic pattern was not solely a consequence of changes in the composition of the total mark. Nevertheless, Reflection scores are assessment outcomes and do not directly measure engagement, teamwork, collaboration, critical thinking or workplace competence; the present study therefore cannot conclude that these competencies improved.

Cohort composition also changed substantially over time. Gender, age group, country/region, enrolment type and delivery mode all differed by year, with the largest differences observed for enrolment and delivery mode. Adjustment for measured characteristics preserved the overall association between year and total marks, but the adjusted Distinction/High Distinction model showed substantial attenuation of most year‐specific contrasts. This indicates that differences in cohort composition may partly explain the observed grade patterns. The country/region sensitivity analyses produced a similar overall interpretation, although the 2020 total‐mark contrast was no longer significant after country adjustment.

Several contextual changes may also have contributed to year‐to‐year variation. The subject was renamed and restructured in 2023, the assessment mix changed and delivery patterns varied, including online‐only delivery during COVID‐related restrictions. At the same time, the same subject coordinator remained in place, the main teaching team was broadly stable, core learning outcomes and marking/moderation processes were substantially unchanged and the intended scope of CSBT was stable. These factors strengthen comparability but do not remove the possibility that curriculum, assessment and delivery changes influenced academic outcomes independently of CSBT.

The unadjusted associations between grades and student characteristics should be interpreted descriptively rather than as evidence that CSBT benefited particular groups more than others. Domestic and online students had higher unadjusted proportions of Distinction/High Distinction than international and on‐campus students, and country/region was strongly associated with grade distribution. These differences may reflect cohort composition, academic transition, assessment context, language and other measured or unmeasured factors [23, 24, 25]. The present study was not designed to identify mechanisms underlying these demographic differences.

## 5 Strengths and Limitations

This repeated‐cohort observational study has several strengths. It included 765 student enrolment records across seven annual cohorts and used routinely collected academic data from a real‐world teaching setting. The stable reflection assessment provided a sensitivity outcome across the full study period, while adjusted models accounted for several measured differences in cohort composition. The analyses therefore provide a detailed description of academic outcomes across successive cohorts using a consistent 2019 reference cohort.

Several limitations should be considered. First, the repeated‐cohort observational design included only one reference cohort and no concurrent control group; therefore, a stable pre‐implementation trend could not be estimated and causal effects of CSBT cannot be inferred. Second, assessment types and weightings changed over time, particularly after the 2023 restructure, so total marks were not based on an identical assessment architecture in every cohort, although the stable Reflection assessment was analysed separately. Third, cohort composition, subject structure and delivery mode changed over time, including online‐only delivery during COVID‐related restrictions; adjustment reduces but does not eliminate confounding. Fourth, the study directly measured academic marks and grades only. It did not independently measure engagement, critical thinking, teamwork, collaboration, decision‐making, workplace competence or employability and no conclusions about changes in these competencies can be drawn. Fifth, students who remained enrolled but did not complete all assessments were retained because their resulting low final marks represented genuine academic outcomes; this contributed to some extreme negative residuals. Finally, qualitative student feedback was not available to explain the mechanisms underlying the observed patterns. Future prospective studies should use comparable assessment structures, concurrent comparison groups where feasible and validated direct measures of educational competencies.

## 6 Conclusion

Across 2019–2025, academic outcomes differed across successive cohorts in a postgraduate public health subject using CSBT. Several later cohorts had higher total marks than the 2019 reference cohort, and the overall association between year and total marks remained after adjustment for measured student characteristics. Grade distributions also varied across years, but the pattern was non‐linear and most adjusted year‐specific Distinction/High Distinction contrasts were not significant. These findings support continued evaluation of CSBT as a teaching approach, but they do not establish that CSBT caused the observed changes or that it improved engagement, critical thinking, teamwork, decision‐making or employability. Future studies using stronger comparative designs and direct validated measures are needed to assess these outcomes.

## Funding

The authors have nothing to report.

## Ethics Statement

This study was reviewed and approved by the Health and Medical Human Research Ethics Committee at the University of Wollongong (Ethics Number: H2026‐0120).

## Conflicts of Interest

The authors declare no conflicts of interest.

## Data Availability

The data used in this study are not publicly available due to privacy, ethical and institutional restrictions related to student records. The data were used under institutional approval for the purposes of this study and cannot be shared publicly.
